# WOX3 in the scene: intimacy with hormones

**DOI:** 10.1093/jxb/erw073

**Published:** 2016-03-07

**Authors:** Million Tadege

**Affiliations:** Department of Plant and Soil Sciences, Institute for Agricultural Bioscience, Oklahoma State University, 3210 Sam Noble Parkway, Ardmore, OK73401, USA

**Keywords:** Dwarfism, electrophoretic mobility shift assay, gibberellic acid, *KAO*, negative feedback regulation, *OsWOX3A*, rice.

**How do WOX genes function to regulate key developmental programs in plants? We’re aware of their love affair with hormones, but lack good evidence of direct cause-and-effect relationships linking transcriptional activity and hormonal changes. In this issue of *Journal of Experimental Botany* (pages 1677–1687), Cho *et al.* provide that evidence for rice OsWOX3A and gibberellic acid.**

The name WOX stands for *WUSCHEL*-related homeobox, named after the founding member of the group, Arabidopsis *WUSCHEL* (*WUS*) (Mayer *et al.*, 1998). WOX transcription factors are plant-specific, homeodomain-containing transcriptional regulators known to function in several key plant developmental programs including embryo development, root and shoot apical meristem maintenance, lateral root and crown root development, tillering and vegetative growth, leaf blade development, vascular patterning, inflorescence development, floral organ development and seed development. A major outstanding question is how WOX genes function to regulate these important processes.

WOX genes do appear to have a love affair with hormones since auxin, cytokinin, abscisic acid (ABA) and gibberellic acid (GA) have been implicated in their actions. However, most of these associations were inferred based on gene expression changes and fluorescent markers signaling changes in the activity of the corresponding hormones. Direct cause-and-effect relationships that demonstrate transcriptional activity of WOX genes linked to measured steady-state level hormonal changes, with classical reversal of effects with hormone applications, have not been well established.

Now, Cho *et al.* (2016) have demonstrated that rice OsWOX3A is induced by GA and directly binds to the promoter of *ent*-kaurenoic acid oxidase (KAO), an enzyme involved in GA biosynthesis, repressing its activity. Transgenic rice plants overexpressing OsWOX3A became dwarf (Fig. 1), suggesting a defect in GA biosynthesis or signaling. By quantifying endogenous GA intermediates, Cho and colleagues were able to show that GA_20_ and GA_1_ levels decrease in these plants. Although *in vivo* data were not provided for the binding of OsWOX3A to the KAO promoter and other potential OsWOX3A binding sites in the GA pathway are still unclear, the induction of OsWOX3A by GA_3_ treatment, reversion of the dwarf phenotype in OsWOX3A transgenic plants by GA application, the measurement of GA intermediates and the interaction of OsWOX3A with the KAO promoter using yeast one-hybrid and EMSA assays are powerful pieces of evidence that together speak loudly for the involvement of OsWOX3A in gibberellin negative-feedback regulation. But then what is the contribution of GA to the *Oswox3a* (*nal2/3*) mutant phenotypes? What other hormones are involved in generating the pleiotropic defects?

**Fig. 1.**
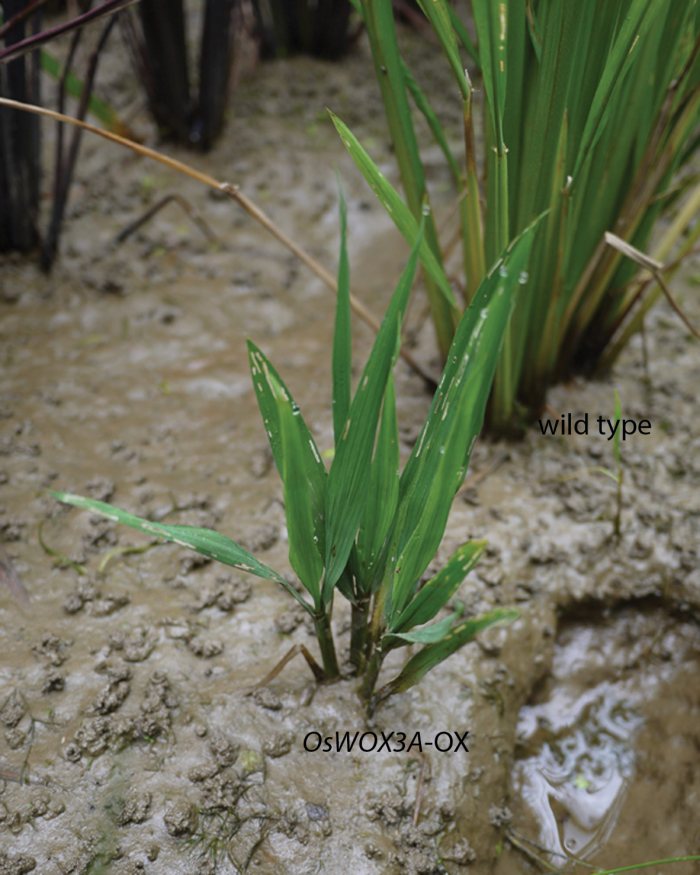
*OsWOX3A*-OX transgenic plants in the field. Courtesy of Prof. Nam-Chon Paek.

## Leaf blade development

Despite the conservation of most Arabidosis WOX genes in eudicots and monocots, *WOX1* and *WOX3* show an interesting detachment. Phylogenetic analyses revealed that Arabidopsis and most eudicots have at least one copy of the *WOX1* and *WOX3* homologs, but grasses have no copy of the *WOX1* homolog at all (Nardmann *et al.*, 2007; Vandenbussche *et al.*, 2009; Tadege *et al.*, 2011; Zhang *et al.*, 2014).

Functionally, Arabidopsis *WOX3 –* also called *PRESSED FLOWER* (*PRS*) – has been proposed to regulate lateral axis-dependent development of flowers (Matsumoto and Okada, 2001), in which the *prs* mutant shows defects or loss in lateral sepals, lateral stamens, and stipules (Nardmann *et al.*, 2004). We now know that *WOX1* and *PRS* function redundantly to regulate leaf blade outgrowth in Arabidopsis (Vandenbussche *et al.*, 2009; Nakata *et al.*, 2012). The *wox1* mutant alone has no obvious leaf phenotype under standard growth conditions but the *wox1 prs* double mutant displays narrow leaf blades. However, mutants in *WOX1* homologs in other dicot species show strong leaf blade and petal fusion phenotypes as single mutants. These mutants were variously named based on their mutant phenotypes, including *maewest* (*maw*) in petunia (Vandenbussche *et al.*, 2009), *stenofolia* (*stf*) in *Medicago truncatula* (Tadege *et al.*, 2011), *bladeless lam1* in *Nicotiana sylvestris* (McHale, 1992; Tadege *et al.*, 2011), *lathyroides* (*lath*) in pea and *narrow organs1* (*nao1*) in *Lotus* (Zhuang *et al.*, 2012). Although some variations exist in the strength of the mutant phenotypes, they all have narrow leaf blades and narrow petals in common. In *stf* and *lam1* mutants where phenotypes are strongest, blades are drastically reduced to less than a third of the wild-type width and carpels are unfused leading to protrusion of ovules and female sterility.

## Promotion of cell proliferation with transcriptional repression

In *Medicago*, *STF* is specifically expressed in leaf primordia in a narrow range of cells in the leaf margin and middle mesophyll at the adaxial–abaxial juxtaposition (Tadege *et al.*, 2011). This type of expression pattern is thought to represent a third (middle) leaf domain (Nakata *et al.*, 2012) between the well-characterized adaxial and abaxial domains. STF/LAM1/WOX1 promotes cell proliferation at the leaf margin, which is mediated by a transcriptional repression activity that involves the transcriptional co-repressor TOPLESS (TPL) (Lin *et al.*, 2013; Zhang *et al.*, 2014). It is, thus, likely that STF/WOX1 maintains a cell proliferation zone at the leaf margin by repressing polarity factors. According to this model, *STF* at the adaxial–abaxial junction provides new cells that ultimately differentiate into adaxial and abaxial domains. This highlights a novel mechanism for blade outgrowth, notwithstanding the well-defined functions of polarity factors (Kerstetter *et al.*, 2001; McConnell *et al.*, 2001). The genetic data are consistent with WOX1 being a repressor of polarity factors (Nakata *et al.*, 2012) and a recent report demonstrated that STF can directly repress the adaxial factor *ASYMMETRIC LEAVES2* (*AS2)* at the margin (Zhang *et al.*, 2014). Because *WOX1* is redundant with *PRS* in Arabidopsis leaf blade outgrowth, but *WOX1* homologs in other dicots independently regulate blade outgrowth, the role of *PRS*/*WOX3* has at least been investigated in *Medicago*. Loss-of-function mutation in *Mtwox3* causes a *loose flower* (*lfl)* phenotype affecting petal fusion (Niu *et al.*, 2015) but no effect on leaf blade outgrowth. This suggests that *STF* is independently recruited for leaf blade outgrowth functions in eudicots, despite the *WOX1* and *PRS* redundancy in Arabidopsis.

In maize, there is no *STF/WOX1* homolog but two *WOX3* homologs, *NARROW SHEATH1* (*NS1*) and *NARROW SHEATH2* (*NS2*), redundantly regulate blade outgrowth (Nardmann *et al.*, 2004). Similarly in rice, two *WOX3* duplicate genes, *NARROW LEAF2* (*NAL2*) and *NARROW LEAF3* (*NAL3*), encoding the same protein OsWOX3A, regulate leaf blade outgrowth (Cho *et al.*, 2013; Ishiwata *et al.*, 2013). The *nal2/3* double mutant displays a pleiotropic phenotype including narrow curly leaves, more tillers, fewer lateral roots, opened spikelets and narrow, thin grains (Cho *et al.*, 2013), indicating a wider effect on overall plant growth and development. Thus, NS/OsWOX3A is the monocot equivalent of STF/WOX1 required for leaf blade outgrowth. Whether OsWOX3A mainly functions as a repressor and recruits OsTPL for this purpose is not known but the results of Cho *et al.* (2016) demonstrate that OsWOX3A represses KAO to maintain GA homeostasis during rice growth and development.

## A sizzling affair with hormones

STF/LAM1 has been proposed to modulate multiple hormone homeostasis and sugar metabolism as part of a mechanism in its regulation of leaf blade outgrowth. Apart from changes in hormone-related gene expression, significantly lower levels of free IAA and ABA have been reported and a similarly low level of free cytokinin has been proposed in *stf* and *lam1* mutants (Tadege *et al.*, 2011), suggesting that STF directly or indirectly affects at least auxin, cytokinin and ABA homeostasis and/or signaling. However, these effects of STF appear to increase hormone levels probably by repressing hormone-conjugating enzymes and/or repressing hormone biosynthesis repressors, but OsWOX3A appeared to directly repress GA biosynthesis. Does this mean that STF and OsWOX3A oppositely affect hormones and the mechanisms for leaf blade development are drastically different in dicots and grasses? Probably not. I imagine that we are just scratching the surface. STF and OsWOX3A are likely to coordinate the effects of several hormones in multiple pathways, but more data are required to pinpoint the similarities and differences.
